# A Novel 5-Enolpyruvylshikimate-3-Phosphate Synthase from *Rahnella aquatilis* with Significantly Reduced Glyphosate Sensitivity

**DOI:** 10.1371/journal.pone.0039579

**Published:** 2012-08-03

**Authors:** Ri-He Peng, Yong-Sheng Tian, Ai-Sheng Xiong, Wei Zhao, Xiao-Yan Fu, Hong-Juan Han, Chen Chen, Xiao-Fen Jin, Quan-Hong Yao

**Affiliations:** Shanghai Key Laboratory of Agricultural Genetics and Breeding, Biotechnology Research Institute, Shanghai Academy of Agricultural Sciences, Shanghai, People's Republic of China; The Scripps Research Institute and Sorrento Therapeutics, United States of America

## Abstract

The 5-enolpyruvylshikimate-3-phosphate synthase (EPSPS; EC 2.5.1.19) is a key enzyme in the shikimate pathway for the production of aromatic amino acids and chorismate-derived secondary metabolites in plants, fungi, and microorganisms. It is also the target of the broad-spectrum herbicide glyphosate. Natural glyphosate resistance is generally thought to occur within microorganisms in a strong selective pressure condition. *Rahnella aquatilis* strain GR20, an antagonist against pathogenic agrobacterial strains of grape crown gall, was isolated from the rhizosphere of grape in glyphosate-contaminated vineyards. A novel gene encoding EPSPS was identified from the isolated bacterium by complementation of an *Escherichia coli* auxotrophic aroA mutant. The EPSPS, named AroA*_R.aquatilis_*, was expressed and purified from *E. coli*, and key kinetic values were determined. The full-length enzyme exhibited higher tolerance to glyphosate than the *E. coli* EPSPS (AroA*_E.coli_*), while retaining high affinity for the substrate phosphoenolpyruvate. Transgenic plants of AroA*_R.aquatilis_* were also observed to be more resistant to glyphosate at a concentration of 5 mM than that of AroA*_E.coli_*. To probe the sites contributing to increased tolerance to glyphosate, mutant *R.aquatilis* EPSPS enzymes were produced with the c-strand of subdomain 3 and the f-strand of subdomain 5 (Thr38Lys, Arg40Val, Arg222Gln, Ser224Val, Ile225Val, and Gln226Lys) substituted by the corresponding region of the *E. coli* EPSPS. The mutant enzyme exhibited greater sensitivity to glyphosate than the wild type *R.aquatilis* EPSPS with little change of affinity for its first substrate, shikimate-3-phosphate (S3P) and phosphoenolpyruvate (PEP). The effect of the residues on subdomain 5 on glyphosate resistance was more obvious.

## Introduction

The enzyme 5-enolpyruvylshikimate-3-phosphate synthase (EPSPS; EC 2.5.1.19) is a carboxyvinyl transferase that catalyzes the transfer of the enolpyruvyl moiety of phosphoenolpyruvate (PEP) to the 5-hydroxyl of shikimate-3-phosphate (S3P) and forms enolpyruvylshikimate-3-phosphate. It is the sixth key enzyme in the shikimate pathway, which is essential for the production of aromatic amino acids Phe, Tyr, and Trp, as well as chorismate-derived secondary metabolites in plants, fungi, and microorganisms [1]. EPSPS has attracted significant attention since it was identified as the primary target of glyphosate in the 1980s [2]. Competitive with PEP, glyphosate forms a stable ternary complex with the enzyme and S3P [3], [4]. The safety and efficacy of glyphosate, together with the widely used glyphosate-resistant crop plants containing a transgene for EPSPS, have combined to make glyphosate the most used herbicide in the world. Herbicide-resistant corn (*Zea mays* L.), canola (*Brassica napa* L.), cotton (*Gossypium hirsutum* L.), alfalfa (*Medicago sativa* L.), and soybeans account for about 82% of biotech crops.

In the past years, a number of glyphosate-resistant bacteria and glyphosate-tolerant plants have been identified. EPSPS from different organisms has been divided into two classes according to intrinsic glyphosate sensitivity. Class I EPSPS was generally identified from plants and bacteria, such as *Escherichia coli* and *Salmonella typhimurium*, the catalytic activity of which was inhibited at low micromolar concentrations of glyphosate [5]. However, the tolerance to glyphosate can be generated by different mutations [6], [7], [8]. For example, alterations of Gly96Ala in *E. coli* and Pro101Ser in *S. typhimurium* have been shown to present resistance to glyphosate. Class II EPSPS, identified from some bacteria, such as *Pseudomonas* strain PG2982, *Agrobacterium tumefaciens* strain CP4, *Streptococcus pneumoniae*, and *Staphylococcus aureus*, was distinguished by its capability to sustain efficient catalysis in the presence of high glyphosate concentrations [9], [10]. These two types of EPSPS share less than 30% amino acid sequence identity [11].

In vineyards, glyphosate is widely used to control weeds directly beneath the vines. Soil and rhizosphere microorganisms from vineyards can harbor highly glyphosate-tolerant EPSPS. In this study, we have isolated a glyphosate-tolerant *Rahnella aquatilis* strain from the rhizosphere of grape in glyphosate-contaminated vineyards. The strain showed a significant inhibition to the pathogen causing crown galls, a worldwide plant disease in grape-growing regions [12], [13]. The isolation, cloning, and characterization of the *R. aquatilis* strain EPSPS, AroA*_R.aquatilis_*, are described. Although this EPSPS shares 88% amino acid identity with the EPSPS (P07638) from *E. coli*, it has more excellent kinetic qualities as it possesses similar binding affinity for the natural substrate PEP but lower affinity sensitivity to glyphosate than the *E. coli* EPSPS. The protein also confers glyphosate tolerance on transgenic plants in greenhouse. Using site-directed mutagenesis, two regions at subdomains 3 and 5 were identified to contribute to increased tolerance to glyphosate.

## Results

### Isolation and identification of the glyphosate-tolerant strain

After selection on low-concentration glyphosate and further verification on high-concentration glyphosate, one strain has been isolated from the rhizosphere of grape. This strain, named GR20, grew well on M9 plates in the presence of 200 mM glyphosate. GR20 was identified as *R. aquatilis* by the biolog identification system. It was small, rod shaped, and Gram negative, and the biochemical test results showed that it was negative for cytochrome oxidase and positive for catalase. The 16S rDNA gene sequence (1,506 bp) confirmed that GR20 was strongly related to *R. aquatilis* (99.8%).

### Cloning and Sequence analysis of the EPSPS gene from *R. aquatilis* GR20

To isolate the gene involved in glyphosate tolerance from *R. aquatilis* GR20, a genomic library was constructed from this strain. There were about 2×10^5^ ampicillin-resistant clones on the LB plates. Selection on M9 plates containing 60 mM glyphosate led to the identification of five clones. The plasmid of all these clones was extracted and digested with *Eco*RI and *Hin*dIII. The clone harboring a plasmid pGR20-5 with about 2.1 kb insert fragment was selected to be sequenced subsequently.

The 2.1 kb insert fragment in pGR20-5 was sequenced and analyzed. A complete open reading frame of 1284 bp with a G + C content of 51.4% was deduced and named AroA*_R. aquatilis_* (GenBank sequence accession number GQ499276). To determine whether ORF427 was translated, AroA*_R. aquatilis_* was expressed and purified. The translated protein of ORF427 was determined to be 47 kDa by sodium dodecyl sulfate-polyacrylamide gel electrophoresis (PAGE), which agreed well with the molecular size of the predicted product (Fig. S1). The N-terminal amino acid sequence of AroA*_R. aquatilis_* was further determined as M-E-S-L-T-L-Q-P-V-A by automated Edman degradation. The result confirmed that the initiator codon of AroA*_R. aquatilis_* is GUG.

**Figure 1:**
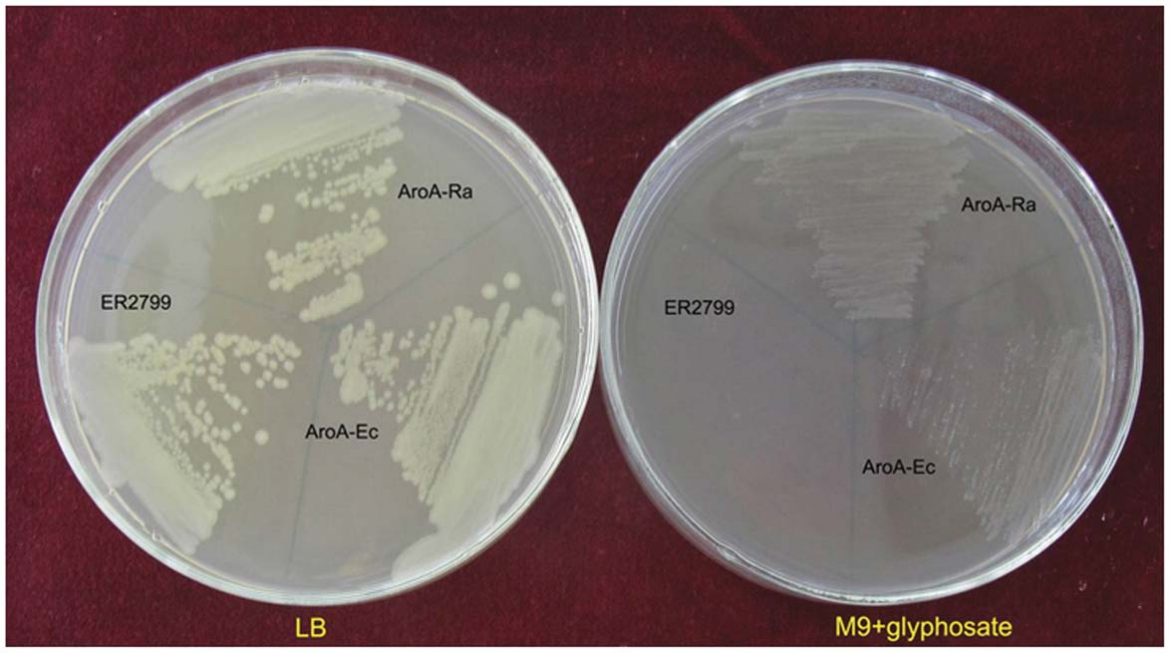
Functional analysis of the *aroA* gene of *R. aquatilis* G20. *In vivo* complementation of the *E. coli* aroA mutant strain ER2799 by the constructs of the AroA fragments. The strain harboring pYPX251, p251-AroA-Ra, and p251-AroA-Ec were tested for growth on LB or M9 minimal medium agar plates with 60 mM glyphosate.

To determine whether ORF427 is sufficient for glyphosate tolerance, a PCR experiment was carried out to amplify only the sequence encoding ORF427 from pGR20-5. The amplified fragment was cloned into the expression vector pYPX251 and transformed into the *E. coli* aroA mutant strain ER2799. The transformants were capable of complementing its aroA mutation for growth on M9 minimal medium supplemented with 60 mM glyphosate (Fig. 1).

**Figure 2:**
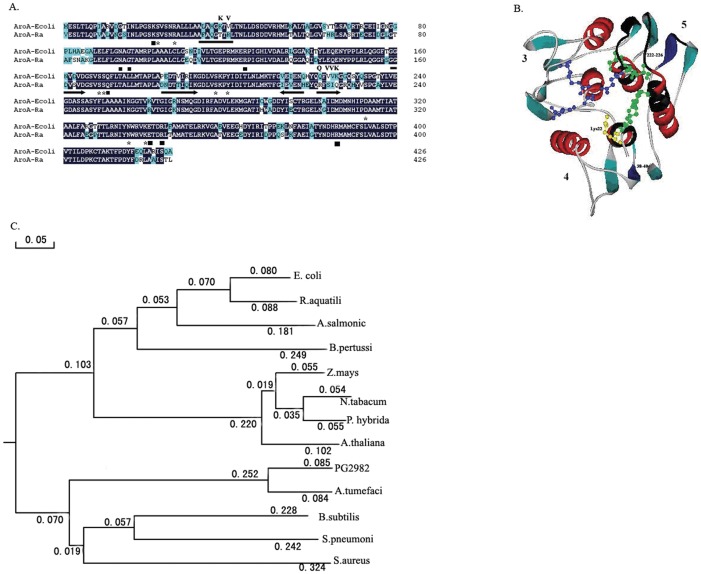
Sequence analysis of the AroA protein from *R. aquatilis* G20. (A) Protein sequence comparison of both AroA protein from *R. aquatilis* G20 and *E. coli*. Amino acids for S3P binding are indicated as asterisks, and amino acids for PEP binding are indicated as squares. The arrow shows the β-sheet in the secondary structure of the subdomain 5. Residues that were mutated in this study were also showed. (B) Views down the axis of the N-terminal domain of the model of AroA*_R.aquatilis_* (AroA-Ra). Each subdomain is numbered according to Stallings et al (30). The proposed S3P and PEP binding residues are rendered in CPK. Residues in chemical shift upon S3P binding are indicated in black. The mutant regions were displayed using blue color. (C) Phylogenetic analysis of AroA constructed using DNAMAN. The tree suggests that AroA*_R.aquatilis_* belonged to Class I AroA protein. Class I AroA proteins were obtained from *E. coli* (P07638), *Aeromonas salmonicida* (Q03321), *Arabidopsis thaliana* (P05466), *N. tabacum* (P23981), *Petunia hybrida* (P11043), *Z. mays* (CAA44974), and *Bordetella pertussis* (P12421). Class II AroA proteins were obtained from *Pseudomonas* strain PG2982 (P56952), *A. tumefaciens* strain CP4 (Q9R4E4), *Bacillus subtilis* (P20691), *S. aureus* (Q05615), and *S. pneumoniae* (Q9S400).

Nucleotide sequence analysis indicated that AroA*_R. aquatilis_* shared more than 80% amino acid identity with *E. coli* EPSPS (AroA*_E.coli_*; Fig. 2A). Detected from the structure of AroA*_R.aquatilis_* modeled by SWISS-MODEL based on the structures of AroA E. coli (PDB 1G6S), the most different amino acids between AroA*_R. aquatilis_* and AroA*_E.coli_* were lined on four-stranded β-sheet structures (159–165; 184–193; 213–218; 221–226) in subdomain 5 [14] and the c-strand of subdomain 3 (Fig. 2B). Phylogenetic tree analysis indicated that AroA*_R. aquatilis_* belonged to class I EPSPS (Fig. 2C).

### Growth of cells in the presence of glyphosate

**Figure 3:**
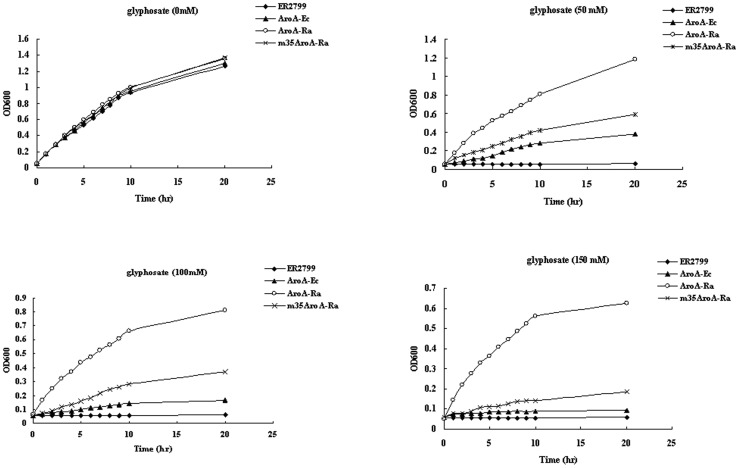
Growth curves of the *E. coli* aroA mutant strain ER2799 cells expressing AroA*_R.aquatilis_* (AroA-Ra), m35AroA*_R.aquatilis_* (m35AroA-Ra, and AroA*_E.coli_* (AroA-Ec) in liquid M9 minimal medium supplemented with glyphosate at the concentrations indicated. The results presented are the averages of two sets of experiments done in triplicate.

The growth curves of the *E. coli* aroA-deleted mutant ER2799 harboring plasmid p251-AroA-Ra or p251-AroA-Ec are shown in Fig. 3. Cells were grown in liquid M9 minimal medium containing various concentrations of glyphosate. The results show that the growth of cells harboring p251-AroA-Ec was strongly inhibited by 50 mM glyphosate. In contrast, cells harboring p251-AroA-Ra can grow at 150 mM glyphosate. The result suggests that AroA*_R. aquatilis_* was more highly tolerant to glyphosate than AroA*_E.coli_.* May be at least one sequence domain at the different regions of subdomain 5 and subdomain 3 is related with glyphosate resistance activity. To determine why AroA*_R aquatilis_* had a higher glyphosate tolerance than AroA*_E.coli_*, the domains of c-strand of subdomain 3, which is near the hinge region for the relationship of the EPSPS two globular structure, and the f-strand of subdomain 5, which was induced to chemical shift changes upon S3P binding, were selected to examine. The residues in the two domain of AroA*_R.aquatilis_* were changed into the corresponding amino acids of AroA*_E.coli_* by multiple-site mutagenesis method (Thr38Lys and Arg40Val, Arg222Gln, Ser224Val, Ile225Val, and Gln226Lys). Cells expressing the regions mutant m35 AroA*_R.aquatilis_* grew much worse than the cells expressing the wild type. The growth potential of the cells expressing m35 AroA*_R.aquatilis_* was similar to that of cells expressing AroA*_E.coli_* in the presence of 100–150 mM glyphosate (Fig. 3).

### Assay for glyphosate tolerance of transgenic tobacco

**Figure 4:**
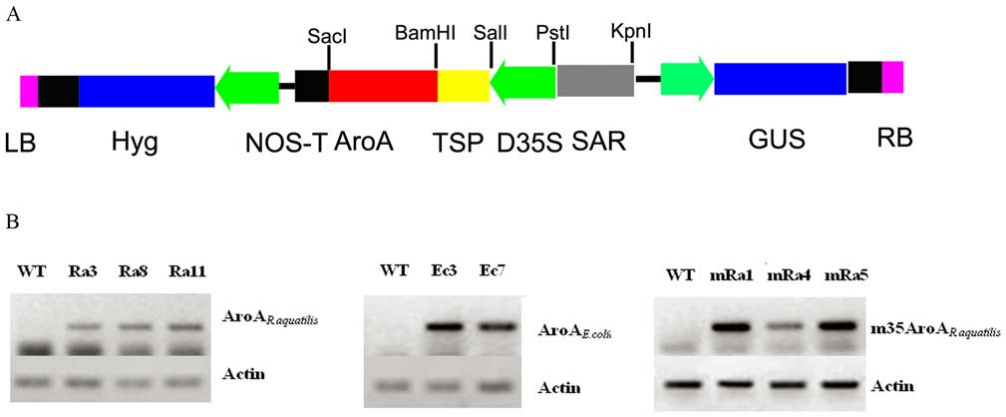
(A) Schematic diagram of the T-DNA region of the binary vectors used in this study. LB, left border; Hyg, hygromycin phosphotransferase gene; D35S, double cauliflower mosaic virus 35S promoter; TSP, tobacco transit peptide; AroA, EPSPS gene; NOS-T, nopaline synthase gene terminator; RB, right border. For the steady transmission of the *aroA* gene, scaffold attachment region (SAR) was fused upstream of the D35S promoter. (B) Evaluation of the glyphosate resistance of transgenic lines. (A) RT-PCR analysis of the transcripts of the *AroA_R.aquatilis_*, *m35AroA_R.aquatilis_*, and *AroA_E.coli_* genes in transgenic and nontransgenic tobacco lines. Normalized expression of the *Actin* gene was used as control. WT, nontransgenic tobacco; Ra3, Ra8, and Ra11, *AroA_R.aquatilis_* transgenic lines; Ec3 and Ec7, *AroA_E.coli_* transgenic lines; mRa1, mRa4, mRa5, *m35AroA_R.aquatilis_* transgenic lines.

Further examine the premise that the AroA*_R.aquatilis_* is better in terms of glyphosate resistance than AroA*_E.coli_* was confirmed in transgenic tobacco. Expression cassettes comprising a CaMV35S promoter, chloroplast transit sequence of tobacco, and EPSPS coding region were cloned into the T-DNA region of the binary vector pYF7716 [15] (Fig. 4A). Then *aroA genes* were introduced to tobacco plants via *Agrobacterium*-mediated transformation. Transgenic plants were obtained from plantlets regenerated on medium containing 30 mg/L hygromycin and further confirmed by PCR analysis. Sixteen AroA*_R.aquatilis_* transgenic plants survived after spraying twice with 5 mM glyphosate. Three AroA*_R.aquatilis_* transgenic lines (Ra3, Ra8, and Ra11) and two AroA*_E.coli_* transgenic lines (Ec3 and Ec7) were analyzed for gene expression using RT-PCR analysis. The results of RT-PCR showed that the inserted genes were actively and stably transcribed in transgenic plants (Fig. 4B).

**Figure 5:**
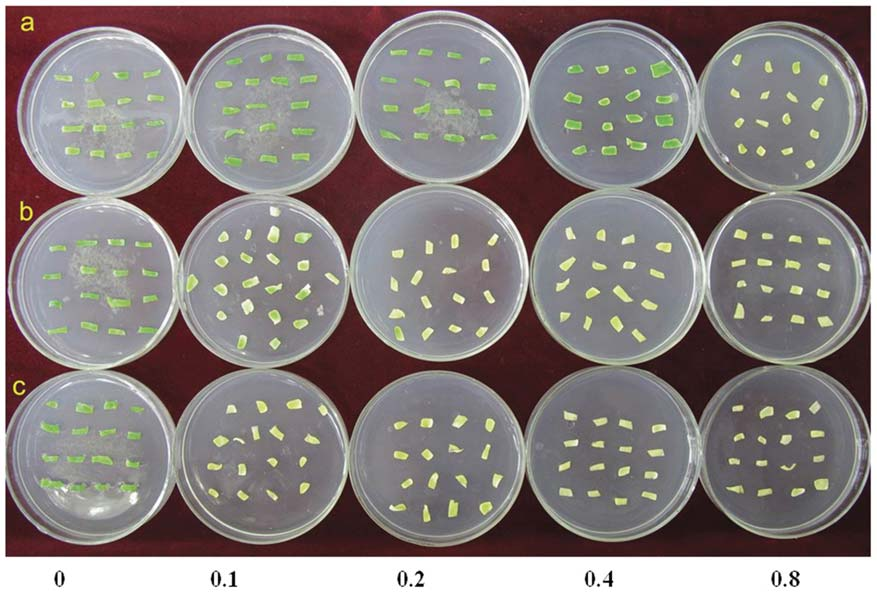
Herbicide resistance in leaf discs after 3 weeks in culture with glyphosate. Leaf discs from transgenic plants *AroA_R.aquatilis_* (a), *AroA_E.coli_* (b), and nontransformed control plants (c) were cultured on solid MS medium containing 0 to 0.8 mM glyphosate (columns 1–5 contained 0, 0.1, 0.2, 0.4, and 0.8 mM glyphosate, respectively).

**Figure 6:**
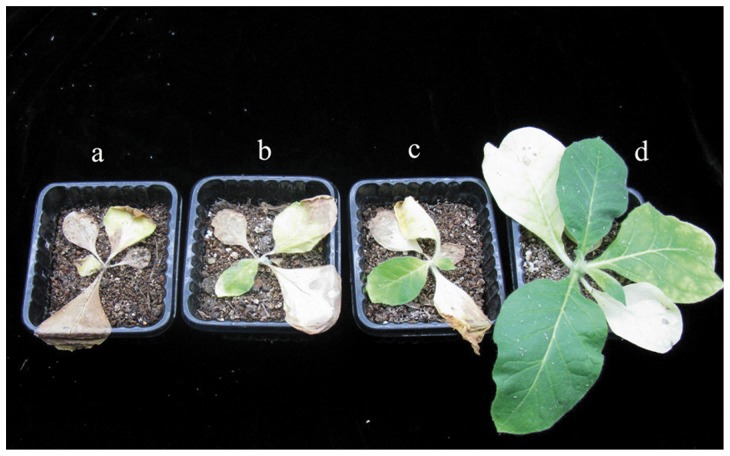
The transgenic plants were sprayed with glyphosate at a dose of 5 mM. Injury was observed visually 2 weeks after the application. (a) nontransformed plants; (b) transgenic tobacco line Ec3; (c) transgenic tobacco line mRa1; and (d) transgenic tobacco line Ra11. For (B) and (C), the same results were obtained in three independent experiments and are represented by the effects shown here.

Subsequently, the glyphosate resistance of transgenic plants was compared at different growth stages. The leaf disc of the transgenic line containing AroA*_R.aquatilis_* had no visible damage at a concentration of 0.4 mM glyphosate, whereas the leaf disc of the transgenic lines containing AroA*_E.coli_* was mortified seriously on the periphery. Untransformed control plants died completely at a concentration of 0.1 mM (Fig. 5). The transgenic lines of AroA*_R.aquatilis_* and AroA*_E.coli_* were also used to test glyphosate resistance by spraying with 5 mM glyphosate in five- to six-leaf stage. AroA*_R.aquatilis_* transgenic plants grew well with normal morphology except for chlorosis of old leaves, whereas AroA*_E.coli_* transgenic plants showed severe chlorosis on most leaves, and nontransformed plants showed very rapid chlorosis and bleaching and then wilted and died (Fig. 6). These results indicate that AroA*_R.aquatilis_* was more resistant to glyphosate exposure than AroA*_E.coli_*, and the characteristic of glyphosate resistance can be heritable in transgenic plants.

To prove both identified regions was critical for glyphosate resistance in class I EPSPS, the mutant, m35AroA*_R.aquatilis_*, was also transfer to tobacco. After examined by RT-PCR (Fig. 4B), the transgenic lines of AroA*_R.aquatilis_* were further assessed for resistance to glyphosate by spraying with glyphosate. In comparison, all transgenic plants containing m35AroA*_R.aquatilis_* have a serious injury as transgenic plants containing AroA*_E.coli_* after two spraying (Fig. 6).

### Kinetic properties of AroA*_R.aquatilis_*

Sequence analysis showed that it was significantly different between AroA*_R.aquatilis_* and AroA*_E.coli_* in the subdomains 3 and 5 of the N-terminal domain. Biological experimentation in transformed *E. coli* and transgenic tobacco plants that compares activity of the constructed mutant gene *m35AroA_R.aquatilis_* with that of the wild-type *AroA_R.aquatilis_* and *AroA_E.coli_* showed that both regions of the c-strand of subdomain 3 (Thr38Lys and Arg40Val) and the f-strand of subdomain 5 are key domains for glyphosate resistance activity.

All mutant and wild EPSPS enzymes were overexpressed separately in *E. coli*, then extracted and purified for activity assay. The *K*_m_ [PEP] and *K*_m_ [S3P] value of AroA*_R.aquatilis_* was similar with that of AroA*_E.coli_*. However, the *K*_i_ [glyphosate] and IC_50_ [glyphosate] of AroA*_R.aquatilis_* values are approximately 35-fold and 5-fold higher than the values of AroA*_E.coli_* respectively (Table 1, Fig. S2). These results exemplify that both enzymes have similar affinity for both substrates PEP and S3P but AroA*_R.aquatilis_* had a higher glyphosate tolerance and a lower level of affinity for glyphosate than AroA*_E.coli_*.

**Table 1:** Kinetic properties of AroA from *R.aquatilis* and *E. coli.* a

Enzyme	Sp act^b^ (U/mg)	*K*_m_ [PEP]^c^ (μM)	*K*_m_ [S3P]^c^ (μM)	*K*_i_ [glyphosate]^d^ (μM)	IC_50_ [glyphosate]^e^ (mM)	*K*_i_ */K*_m_[PEP]
AroA*_R.aquatilis_*	82.8 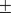 3.1	78.3 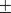 2.3	86.3 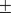 3.5	49.6 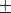 5.2	0.26 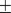 0.02	0.63
m3AroA*_R.aquatilis_*	66.4 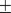 4.4	74.5 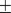 4.5	72.1 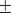 6.2	17.2 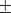 1.9	0.17 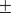 0.01	0.24
m5AroA*_R.aquatilis_*	54.3 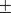 3.2	71.4 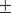 3.6	69.8 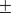 2.1	13.5 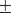 3.6	0.13 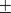 0.01	0.18
m35AroA*_R.aquatilis_*	48.7 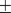 4.1	66.3 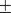 4.2	71.2 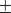 3.8	9.0 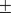 0.7	0.11 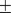 0.01	0.14
AroA*_E.coli_*	41.1 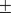 5.3	65.8 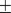 5.6	60.1 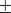 3.4	1.4 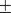 0.1	0.05 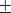 0.01	0.021
m5AroA*_E.coli_*	60.3 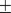 3.7	67.6 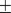 2.9	65.3 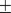 2.9	8.4 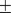 3.4	0.15 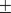 0.01	0.22
_G96A_ AroA*_E.coli_*	71.4 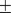 4.5	119.5 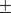 5.1	61.2 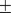 4.5	219.3 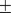 1.9	0.22 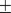 0.02	0.19
_G96A_ AroA*_R.aquatilis_*	100.5 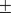 2.9	148.3 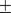 4.5	84.5 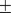 5.4	450.4 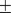 3.4	0.34 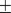 0.01	0.38

a The results presented are the averages of two sets of experiments done in triplicate.

b Sp act, specific activity; 1 unit (U) of EPSPS activity is defined as the amount of enzyme that catalyses the liberation of 1 μmol inorganic phosphate per min reaction time. Determined at 1.0 mM PEP and 1.0 mM S3P.

c Determined by the Lineweaver-Burk method. The PEP or S3P concentration was fixed at 0.05, 0.075, 0.1, 0.2, 0.5, and 1 mM, respectively, while the other concentration was fixed at 1 mM.

d Competitive inhibition by glyphosate with respect to PEP was demonstrated by lines converging on the x axes of Lineweaver-Burk plots. The PEP concentration was fixed at 0.05, 0.1, 0.2, and 0.5 mM, respectively, while the glyphosate concentration was 0, 10, 20, 50, and 100 μM, and the S_3_P concentration was fixed at 1 mM.

e The half maximal inhibitory concentration (IC50) is a measure of the effectiveness of glyphosate in inhibiting biochemical function. It was determined by fitting the data to the equation: *V*  =  *V*_min_ + (*V*_max_−*V*_min_)/(1 + ([*I*]/IC_50_)^s^), and *V* was determined at 1 mM PEP and 1 mM S3P with the glyphosate concentration ranging from 0.0001 mM to 100 mM.

Biochemical analyses of mutants have also allowed some regions to be identified as essential or nonessential for glyphosate resistance activity. Kinetic characterizations of the wild-type AroA*_R.aquatilis_* and its mutants on both regions were detected. The *K*_m_ [PEP] and *K*_m_ [S3P] of the mutant m35AroA*_R.aquatilis_* were similar to the wild type, whereas the values of *K*_i_ [glyphosate] and IC_50_ [glyphosate] decreased apparently respect to the wild type enzyme. The values of *K*_i_ [glyphosate] and IC_50_ [glyphosate] of m35AroA*_R.aquatilis_* are only 6-fold and 2-fold higher than that of AroA*_E.coli_* respectively (Table 1). These results indicated that the c-strand of subdomain 3 and the f-strand of the subdomain 5 were important for glyphosate binding and conferred a close relationship with glyphosate resistance. To detect what is contribution for glyphosate resistance, more kinetic analysis of the independent mutant on both regions was done. The *K*_m_ [PEP] and *K*_m_ [S3P] of the two mutants (m3AroA*_R.aquatilis_* and m5AroA*_R.aquatilis_*) have no significant change. But the values of *K*_i_ [glyphosate] and IC_50_ [glyphosate] decreased when compared with that of the wild type. The ratios of the wild type enzyme over the mutant m3AroA*_R.aquatilis_* were approximately 2.8 and 1.5 for *K*_i_ [glyphosate] and IC_50_ [glyphosate] respectively, while the ratios over the mutant m5AroA*_R.aquatilis_* were 3.7 and 2.0. It indicated that the f-strand of the subdomain 5 was more important for glyphosate resistance than the c-strand of subdomain 3 (Table 1). Further, in order to testify that the f-strand of the subdomain 5 was important for glyphosate binding, we have generated m5AroA*_E.coli_* with the subdomain 5 of AroA*_R.aquatilis._* The kinetic characterizations showed that the values of *K*_i_ [glyphosate] and IC_50_ [glyphosate] of m5AroA*_E.coli_* are 7-fold and 3-fold higher than that of AroA*_E.coli._* It is obvious that the f-strand of the subdomain 5 was indeed important for glyphosate binding.

## Discussion

In this study, a novel *aroA* gene was cloned from an *R. aquatilis* strain isolated from the rhizosphere of grape in glyphosate-polluted vineyards. It was shown that the *aroA* gene product (AroA*_R.aquatilis_*) could complement *E. coli* AroA mutations. Sequence analysis revealed that AroA*_R.aquatilis_* was most closely related to the EPSPS of *E. coli* AroA*_E.coli_* and belonged to class I AroA enzymes (Fig. 2C).

In addition to wastewater, puddle, and human clinical specimens, *R. aquatilis* can also be isolated from nonpolluted soil. Some strains of *R. aquatilis* were plant rhizosphere-associated bacteria. *R. aquatilis* has been reported to be a potential biological control agent for different *Agrobacterium* strains, which are causal organisms of crown gall disease [16]. Although the genus of *Rahnella* is widely distributed in water and soil, nobody has noticed that *R. aquatilis* strain have natural glyphosate tolerance until now. *R. aquatilis* may have acquired an *aroA* gene from other soil microbial communities or improved glyphosate tolerance of AroA enzymes through natural selection.

We have examined the glyphosate resistance activity of AroA*_R.aquatilis_* in transformed *E. coli* and transgenic tobacco. Transformed *E. coli* and transgenic plants with AroA*_R.aquatilis_* showed greater glyphosate resistance as compared to AroA*_E.coli_*. The AroA*_R.aquatilis_* transgenic plant line can survive treatment with 5 mM glyphosate. Kinetic analysis of the AroA*_R.aquatilis_* also indicated that it had lower glyphosate affinities than AroA*_E.coli_*.

The reaction of EPSPS with S3P and PEP and the inhibition of this reaction by glyphosate have been extensively studied during the past 30 years. Glyphosate is a reversible inhibitor of EPSPS because it can bind adjacent to S3P in the PEP binding site and mimic an intermediate state of enzyme–substrates complex. In recent years, a large number of active sites of EPSPS have been studied and identified [17], [18], [19], [20]. The glyphosate binding site is dominated by charged residues Lys22, Arg124, and Lys411, which have previously been concerned in PEP binding [21]. Mutation of these amino acids can result in a significant change in glyphosate tolerance. Target site glyphosate tolerance can also be induced by other specific mutations, including Thr42Met [22], Gly96Ala [23], Thr97Ile [24], Pro101Leu, Pro101Thr, Pro101Ala, and Pro101Ser [8], [18], [25], and Ala183Thr [23], in which residues 97–105 form an internal helix in the N-terminal globular domain and correct with glyphosate closely, and Ala183 is an important residue for EPSPS-glyphosate interaction. The residues important for S3P binding and PEP binding and the domains for glyphosate tolerance were found to be conservative between AroA*_R.aquatilis_* and AroA*_E.coli_*.

As compared to the *E. coli* AroA protein, AroA*_R.aquatilis_* has two significant different regions in the N-terminal domain. Although both different regions were at the position distant from the active site for S3P binding and PEP binding, they are related with the conformational change of N- and C-terminal globular domain. One region is located in subdomain 5. Residues in the four-stranded β-sheet structures (159–165, 184–193, 213–218, and 221–226) of this domain are changed greatly (Fig. 2A and B). Some residues in the f-strand of the subdomain 5 will be induced to chemical shift changes upon S3P binding. As revealed in X-ray crystallography studies, S3P triggers the two globular domains to move toward each other in a screw-like movement and cause its transition from the open to the closed state [21]. These different residues may induce structural adjustments in hydrogen bonding networks and result in positional shifts of the residues connected to the substrate [26]. The other is located in the c-strand of subdomain 3 (Fig. 2A and B). In this region, some residues are near the hinge region, which will alter the relationship of the two globular structures of EPSPS. This proposed mechanism was corroborated with the finding that the substitution of bulkier hydrophobic amino acid methionine for Thr42, a residue near the hinge region, affects the screw-like closure by altering the relative positions of the clusters of the active site amino acids that reside on opposite globular domains [7].

To prove that both regions affected the glyphosate affinities of AroA, the c-strand of subdomain 3 and the f-strand of subdomain 5 of AroA*_R.aquatilis_* were substituted with the corresponding residues in the same region of AroA*_E.coli_* by multiple-site mutagenesis method. Both domain replacements decreased the *K*_i_ [glyphosate] and IC_50_ significantly. However, the *K*_m_ [PEP] and *K*_m_ [S3P] changed trivially. This implied that glyphosate tolerance of AroA was related to the regions containing residues whose chemical shifts change upon S3 binding and the regions containing residues near the hinge domain. Further experiment proved that the f-strand of subdomain 5 contributed more to glyphosate tolerance than the c-strand of subdomain 3 (Table 1, Fig. S2). It suggested that the open conformation of the enzyme-S3P complex will affect the glyphosate affinities.

Although a large number of AroA enzymes have been identified and tested as glyphosate resistant, only AroA variants derived from the *A. tumefaciens* strain CP4 have been successfully used commercially [10]. Many single-site mutations have been made to investigate the molecular basis of the glyphosate insensitivity of the AroA enzyme. However, glyphosate tolerance is often paralleled by a decrease in the affinity of EPSPS for PEP. Thus, they were insufficient for commercial glyphosate-resistant crops. For example, although the *K*_i_ [glyphosate] value of the *E. coli* EPSPS mutant Gly96Ala was more than 1000-fold higher than that of the wild type, the *K*_m_ [PEP] of the mutant increased 23 times [11]. Multisite mutations with more favorable properties were sought and discovered. For example, the double mutant *Zea mays* EPSPS Thr102Ile/Pro106Ser (abbreviated as TIPS) had particularly favorable characteristics. The TIPS mutations have been introduced into the endogenous EPSPS enzyme of *Z. mays* to produce the first commercial varieties of glyphosate-resistant maize (U.S. Patent 6,040,497). In this research, we found that AroA*_R.aquatilis_*, which showed 88% amino acid identity with the AroA from *E. coli*, had greater glyphosate tolerance without decreasing its affinity for PEP and S3P. AroA*_R.aquatilis_* may help to obtain a new type of class I AroA proteins that not only show high tolerance to glyphosate but also have high affinity for PEP. This can be done through domain replacement or multi-sites mutation but not single-site mutation. Through site-directed mutation and optimal codon usage, the new *aroA* gene is likely to improve its efficiency for glyphosate tolerance in engineering crops.

## Materials and Methods

### Medium and chemicals

S3P, glyphosate (free acid form), and PEP were purchased from Sigma-Aldrich. All other chemicals were of analytical grade. *E. coli* strain ER2799 (with the EPSPS gene deleted from its genome) was a kind gift from Professor Thomas C. Evans, Jr. (New England Biolabs, USA). *Agrobacterium* strains were bought from the Agricultural Culture Collection of China.

### Study area and ethical approval

The field studies were conducted in a vineyard which located at Hongqi village, Minheng district, Shanghai, China. Glyphosate has been used extensively as a broad-spectrum control agent to kills weed plants over 10 years in this vineyard.

This study involved collection of 10 samples of soil from the rhizosphere of grape in glyphosate used extensively vineyards, without human participants and animal work, therefore the local ethics committee considered that informed verbal consent was appropriate and this was approved by district officials, village, sub-village leaders and the owner of this vineyards. No specific permits were required for the field studies. Also the field studies did not involve endangered or protected species and no specific permissions were required for these locations. And local ethics committee waived the need for consent.

### Isolation procedure

Isolation of the glyphosate-tolerant strains was carried out with soil samples from the rhizospheres of grape in glyphosate-contaminated vineyard. A pool of soils from the rhizospheres of grape in 10 different sites in this vineyard were washed with 0.9% (w/v) NaCl solution and then spread onto M9 plates containing 60 mM glyphosate. Dozens of colonies were isolated after 2 days of incubation at 28°C and further screened on M9 plates in the presence of 200 mM glyphosate. One well-grown strain, GR20, was chosen for further studies.

### Identification of strain GR20

The morphological, cultural, biochemical, and physiological characteristics of GR20 were examined according to the methods previously reported by Farmer [27]. Chromosomal DNA of GR20 was extracted and purified according to the reported method [28]. The 16S rDNA was amplified from GR20 genomic DNA with the primers 16SR (5′-AGAGTTTGATCCTGGCTCAG-3′) and 16SF (5′-ACGGCTACCTTGTTACGACTTC-3′). The PCR fragment was cloned and sequenced.

### Isolation of the gene involved in glyphosate tolerance

Chromosomal DNA isolated from strain GR20 was partially digested with *Sau*3A, and DNA fragments of 2 to 4 kb were purified and inserted into the *Bam*HI site of dephosphorylated pUC18 [29]. A genomic library was constructed by electroporating ligated DNA into *E. coli* DH5α-competent cells. Then the genomic library plasmid was transformed to the *E. coli* aroA mutant strain ER2799 and screened on M9 agar plates supplemented with 60 mM glyphosate. One colony harboring a plasmid pAroA-Ra5 with about 2.0 kb insert fragment was selected to be sequenced subsequently.

### Gene cloning and mutagenesis

Primers GR20Z2 (5′-GAGAGAGGATCCATGGAATCCCTGACATTACAAC-3′) and GR20F2 (5′-GAGAGAGAGCTCTTATGCCAGTGTGCTGATTGC-3′) were used to amplify the EPSPS gene *AroA_R.aquatilis_* from pAroA-Ra5. Multiple-site mutagenesis was carried out in order to induce mutations in the hinge region (Thr38Lys and Arg40Val) and the subdomain 5 (Arg222Gln, Ser224Val, Ile225Val, and Gln226Lys) of *AroA_R.aquatilis_* by PAGE-mediated overlap extension PCR [30]. The following oligonucleotide pairs were used: M1Z (5′-GCTTTTGCACAGGGTAAAACCGTCCTGACTAACCT GCT CGA-3′) and M1F (5′-TCGAGCAGGTTAGTCAGGACGGTTTTACCCTGTGCAAAAGC-3′) for mutations in the hinge region and M2Z (5′-ACCACGAGTACCAGCAATTTGTCGTCAAAGGA CGTCAGCATTA-3′) and M2F (5′-TAATGCTGACGTCCTTTGACGACAAATTGCTGGTACTCGT GGT-3′) for mutations in subdomain 5. The first PCRs were performed to amplify three mutant fragments using GR20Z and M1F, M1Z and M2F, and M2Z and GR20F. PCR products were resolved using 8% polyacrylamide gels, recovered and purified by electroelution into dialysis bag, and concentrated by ethanol precipitation. The full-length mutant gene *m35AroA_R.aquatilis_* was obtained by PCR with a pair of primer GR20Z and GR20F and the mixed templates combined with the above three purified DNA fragments. Single-site mutagenesis was carried out to obtain the mutant gene *m5AroA_R.aquatilis_* or *m3AroA_R.aquatilis_* through direct amplification from pAroA-Ra5 with the prime M1Z and M1F or M2Z and M2F. In order to further testify that the f-strand of the subdomain 5 was important for glyphosate binding, we have generated m5AroA*_E.coli_* with the subdomain 5 of AroA*_R.aquatili._* Also, the _G96A_AroA*_E.coli_* [31] and _G96A_AroA*_R.aquatilis_* were created using a PCR-based staggered extension process.

### Comparison of cell growth in the presence of glyphosate

Primers EZ1 (5′-CGGGATCCGTTAATGCCGAAATTTTGCTTAATC-3′) and EF1 (5′-CGGAGCT CAGGTCCGAAAAAAAACGCCGAC-3′) were used to amplify the EPSPS gene *AroA_E.coli_* from the chromosomal DNA of *E.coli*. The amplified fragments of the wild type gene *AroA_R.aquatilis_*, mutant gene *m35AroA_R.aquatilis_* and *AroA_E.coli_* were digested with *Bam*HI and *Sac*I and ligated into the pYPX251 vector (GenBank accession number AY178046) to construct recombinant plasmids p251-AroA-Ra, p251-m35AroA-Ra and p251-AroA-Ec, respectively. These EPSPS genes would be driven by the moderate promoter of aacC1.

The *E. coli* aroA-deleted mutant strain ER2799 harboring either p251-AroA-Ra, p251-m35AroA-Ra, and p251-AroA-Ec was grown by shaking at 37 °C in liquid M9 minimal medium supplemented with glyphosate at various concentrations ranging from 0 to 150 mM. Cell densities were measured by attenuation at 600 nm [11].

### Construction of plant expression vector and plant transformation

A DNA fragment encoding the chloroplast transit peptide of tobacco (TSP; GenBank accession number M61904) was PCR amplified from the genomic DNA of tobacco using primers TSPZ (5′-CGCCTGCAGTGGCACAGATTAGCAGCATG-3′) and TSPF (5′-CAGAGGATCCTCTGTGC AGTGACCACTGAT-3′). The PCR fragment was digested with *Sal*I and *Bam*HI, cloned into the corresponding restriction sites of pGEM3Z (Promega, Madison, WI, USA), and confirmed by DNA sequencing. The *Pst*I-*Sal*I fragment of double CaMV35S promoter was inserted upstream of the TSP sequence in the sequence confirmed plasmid. The fused fragment and *aroA* gene were cloned into the *Agrobacterium* binary vector pYF7716 [15] using *Pst*I-*Bam*HI and *Bam*HI-*Sac*I restriction sites, respectively. The final constructs D35S:TSP:aroA:Nos (Fig. 4A) were introduced into *A. tumefaciens* LBA4404 (Clontech, Palo Alto, CA) by electroporation. The tobacco (*Nicotiana tabacum* cv *Xanthi*) was transformed by the leaf discs method via the *Agrobacterium*-mediated transformation procedure described by Wang et al. [32]. Shoots were rooted on a medium containing 30 mg/L hygromycin and then transferred into soil and grown in a greenhouse.

### Transgenic plants selection and assay for herbicide resistance

The transgenic plants were confirmed by PCR with the specific primers GR20Z2 and GR20F2 for *AroA_R.aquatilis_*, *m35AroA_R.aquatilis_* and EZ1 and EF1 for *AroA_E.coli_*. PCR amplification was performed using the genomic DNA of hygromycin-resistant plants as template. RT-PCR was performed to confirm the transcription of the *AroA_R.aquatilis_*, *m35AroA_R.aquatilis_* and *AroA_E.coli_*. Total RNA was extracted from the seedlings of T_1_ generations using an RNA isolation kit (MBI, Fermentas), and first-strand cDNA was synthesized using 5 µg total RNA with the Reverse Transcription System (Promega, Madison, WI, USA). The fragments of the tobacco *Actin* gene were amplified from the same cDNAs as a control to normalize the amount of cDNA using primers TactZ (5′-CAATGAACTTCGTGTGGCTCC-3′) and TactF (5′-CGGAATCTCTCAGCACCAATG-3′). The specific fragment of about 200 bp from *AroA_R.aquatilis_*, *m35AroA_R.aquatilis_* and *AroA_E.coli_* gene was amplified from transgenic plants using the same amount of cDNA. The PCR products were separated on 2% agarose gel and quantified using a Model Gel Doc 1000 (Bio-Rad, USA). The expression pattern of those *aroA* genes was evaluated with a Shine Tech Gel Analyser (Shanghai Shine Science of Technology Co., Ltd., China). The same results were obtained for three independent experiments.

The leaf discs were cut from individual transgenic tobacco and then moved to Murashige and Skoog (MS) medium containing 0 to 0.8 mM glyphosate and grown for 3 weeks with 16 h of light and 8 h of dark. Seedlings of T_1_ generations were transplanted into soil and grown to five- to six-leaf stage. After spraying with the 5 mM glyphosate for 2 weeks, injury of the transgenic plants was observed visually.

### Overexpression and purification of AroA

DNA fragments containing the entire coding region of *AroA_R.aquatilis_* and the mutants *m35AroA_R.aquatilis_*, *m3AroA_R.aquatilis_*, and *m5AroA_R.aquatilis_* was obtained by PCR from the positive colony with the following two primers: GR20Z (5′-GAACCATGGAATCCCTGACATTACAACCCGT-3′) and GR20F (5′-GTTCTCGAGTGCCAGTGTGCTGATTGCCGCCA-3′). Primer GR20Z contains a mutation where the initiation start codon ATG was replaced with GUG. The fragment was digested with *Nco*I and *Xho*I, cloned into the corresponding restriction sites of pET-28a (Novagen, Inc.), and confirmed by DNA sequencing. Then the plasmid was transformed to *E. coli* BL21 (DE3; Novagen, Inc.). The expressed protein was purified using a HisTrap HP kit (Amersham Biosciences) according to the manufacturer's instructions. The AroA*_E.coli_* from *E. coli* was overexpressed and purified according to the method used above. Also the protein of *AroA_R.aquatilis_* was further blotted onto a polyvinylidene difluoride membrane for N-terminal protein sequencing using an Applied Biosystems Procise sequencer.

### Enzyme assay

EPSPS activity was measured by determining the amount of inorganic phosphate produced in the reaction using the malachite green dye assay method [33]. The standard reaction was carried out at 28°C in a final volume of 50 µl containing 50 mM HEPES (pH 7.0), 1 mM S3P, 1 mM PEP, and the purified enzyme. The enzyme was allowed to react for 3 to 5 min before 800 µl malachite green–ammonium molybdate colorimetric solution was added. Color development was stopped after 1 min by addition of 800 µl of a 34% sodium citrate solution. After incubation for 30 min at room temperature, the absorbances of the samples were measured at 660 nm. In this case, the same reaction solution without S3P was used as the zero control.

*K*_m_ values for PEP, *K*_i_ values for glyphosate, and IC50 values (the concentration of glyphosate inhibiting enzyme activity by 50%) were measured as described by Tian et al. [34], [35], [36].

### DNA sequence GenBank Accessions

The novel 1284 bp DNA sequence encoding the 5-enolpyruvylshikimate-3-phosphate synthase from *Rahnella aquatilis* was deposited as GenBank Accession number GQ499276.

## Supporting Information

Figure S1 **Overexpression and purification of AroA** ***_R.aquatilis_*** **.** Lane 1, molecular mass marker; lane 2, protein of *E.coli* ER2799; lane 3, overexpression of AroA*_R.aquatilis_* induced with IPTG; lane 4, purified protein with HisTrap HP kit.
(TIF)

Figure S2 **Kinetic properties of AroA** ***_E.coli_*** **., m35AroA** ***_R.aquatilis_*** **, m3AroA** ***_R.aquatilis_*** **, m5AroA** ***_R.aquatilis_*** **, wild type AroA** ***_R.aquatilis_*** **, m5AroA** ***_E.coli, G96A_*** **AroA** ***_E.coli_ and_ G96_*** **AroA** ***_R.aquatilis._*** A. Steady-state kinetics of AroA*_E.coli_*., m35AroA*_R.aquatilis_*, m3AroA*_R.aquatilis_*, m5AroA*_R.aquatilis_*, wild type AroA*_R.aquatilis_*, m5AroA*_E.coli, G96A_*AroA*_E.coli_* and *_G96_*AroA*_R.aquatilis_* Activities were assayed in Hepes buffer at 28°C in the presence of 1 mM S3P and various concentrations of PEP. B: Steady-state kinetics of AroA*_E.coli_*., m35AroA*_R.aquatilis_*, m3AroA*_R.aquatilis_*, m5AroA*_R.aquatilis_*, wild type AroA*_R.aquatilis_*, m5AroA*_E.coli, G96A_*AroA*_E.coli_* and *_G96_*AroA*_R.aquatilis._* Activities were assayed in Hepes buffer at 28°C in the presence of 1 mM PEP and various concentrations of S3P. C. IC_50_ values of AroA*_E.coli_*., m35AroA*_R.aquatilis_*, m3AroA*_R.aquatilis_*, m5AroA*_R.aquatilis_*, wild type AroA*_R.aquatilis_*, m5AroA*_E.coli, G96A_*AroA*_E.coli_* and *_G96_*AroA*_R.aquatilis._* The IC_50_ values of glyphosate inhibition were determined by fitting the data to the equation *v* = *V_min_*+ (*V_max_*−*V_min_*)/ [1+ ([*I*]/IC_50_)*^s^*], and *v* was determined at 1 mM PEP and 1 mM S3P, with glyphosate concentrations ranging from 0.0001 to 100 mM.
(TIF)
