# Supplementary figures and images for: A Novel 5-Enolpyruvylshikimate-3-Phosphate Synthase from Rahnella aquatilis with Significantly Reduced Glyphosate Sensitivity
Source: PLoS One. 2012 Aug 3;7(8):e39579. doi: 10.1371/journal.pone.0039579 (PMC3411725; doi:10.1371/journal.pone.0039579)

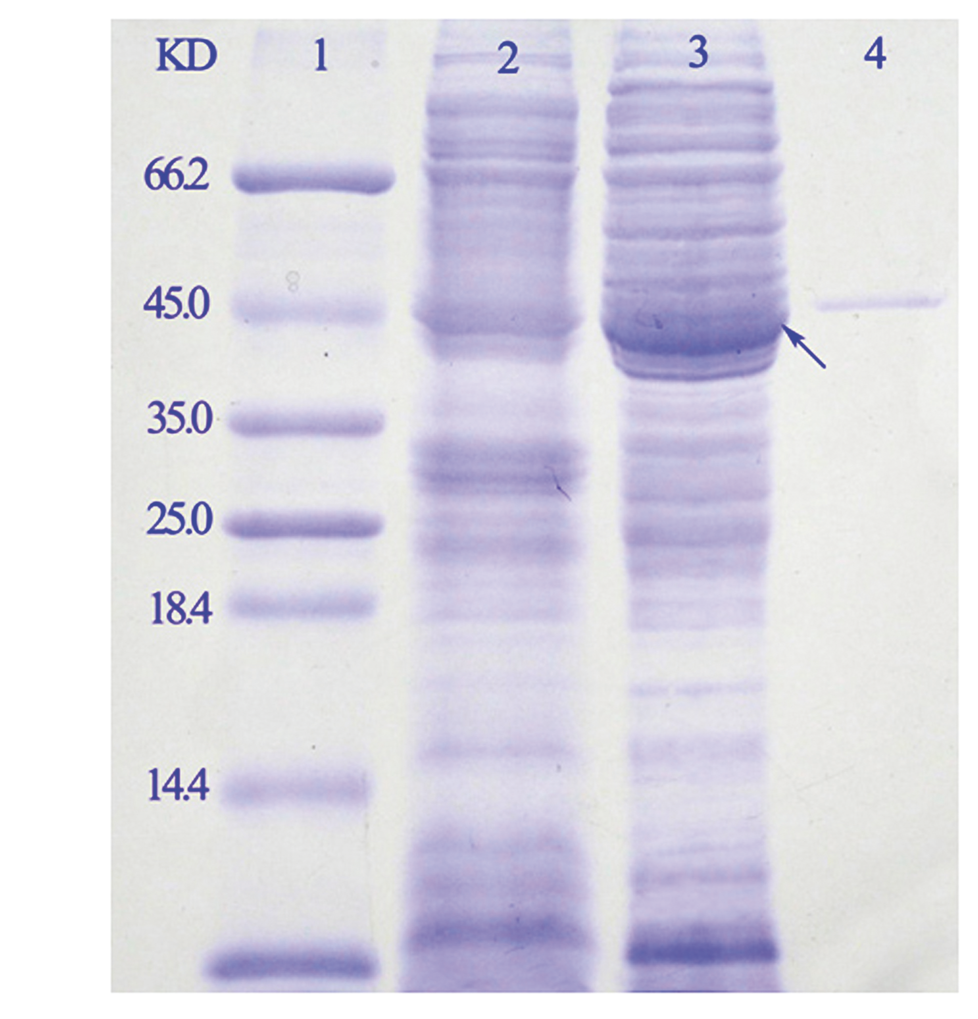

Supplement: Figure S1 — Overexpression and purification of AroA R.aquatilis . Lane 1, molecular mass marker; lane 2, protein of E.coli ER2799; lane 3, overexpression of AroAR.aquatilis induced with IPTG; lane 4, purified protein with HisTrap HP kit. (TIF) [file pone.0039579.s001.tif]

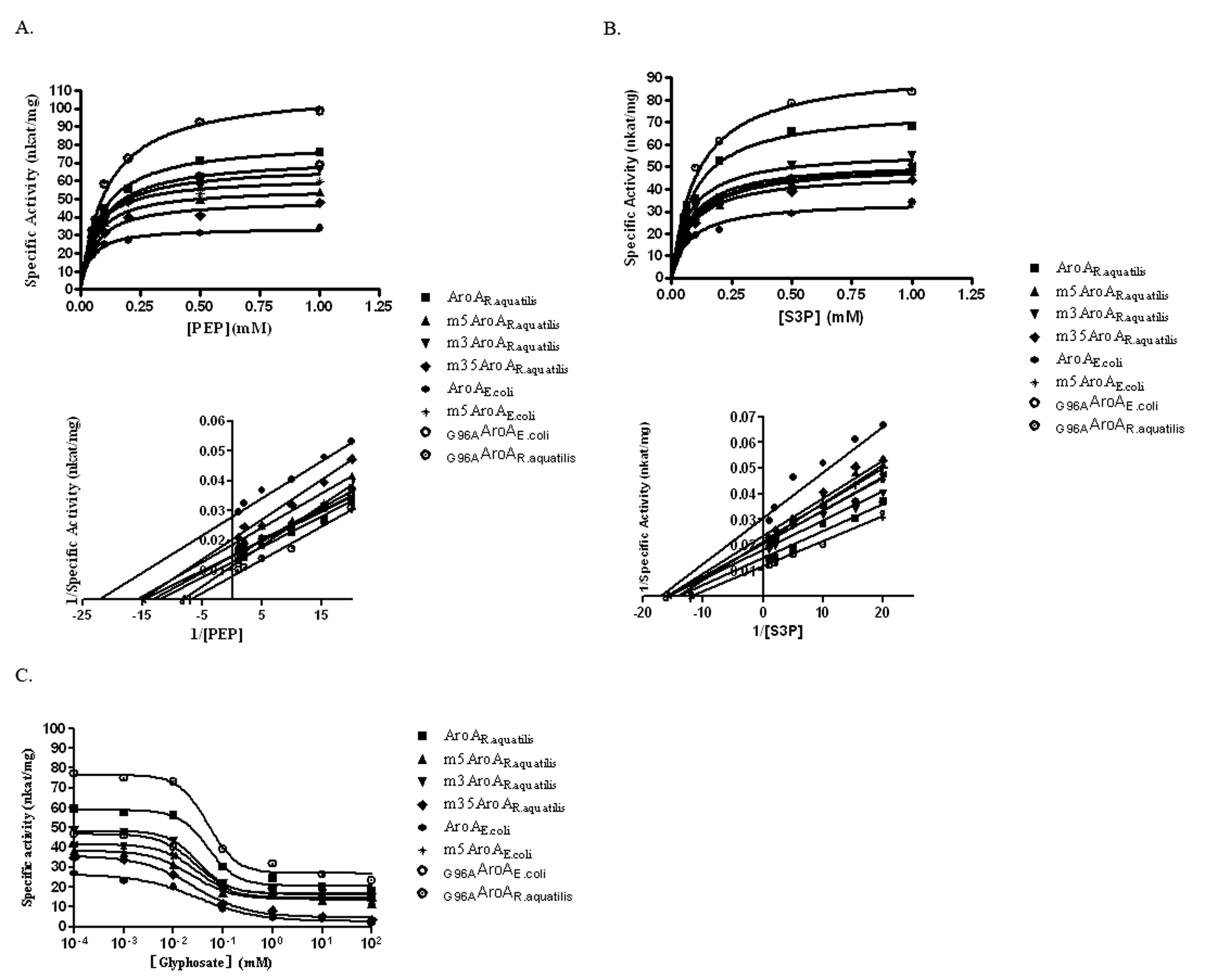

Supplement: Figure S2 — Kinetic properties of AroA E.coli ., m35AroA R.aquatilis , m3AroA R.aquatilis , m5AroA R.aquatilis , wild type AroA R.aquatilis , m5AroA E.coli, G96A AroA E.coli and G96 AroA R.aquatilis. A. Steady-state kinetics of AroAE.coli., m35AroAR.aquatilis, m3AroAR.aquatilis, m5AroAR.aquatilis, wild type AroAR.aquatilis, m5AroAE.coli, G96AAroAE.coli and G96AroAR.aquatilis Activities were assayed in Hepes buffer at 28°C in the presence of 1 mM S3P and various concentrations of PEP. B: Steady-state kinetics of AroAE.coli., m35AroAR.aquatilis, m3AroAR.aquatilis, m5AroAR.aquatilis, wild type AroAR.aquatilis, m5AroAE.coli, G96AAroAE.coli and G96AroAR.aquatilis. Activities were assayed in Hepes buffer at 28°C in the presence of 1 mM PEP and various concentrations of S3P. C. IC50 values of AroAE.coli., m35AroAR.aquatilis, m3AroAR.aquatilis, m5AroAR.aquatilis, wild type AroAR.aquatilis, m5AroAE.coli, G96AAroAE.coli and G96AroAR.aquatilis. The IC50 values of glyphosate inhibition were determined by fitting the data to the equation v = Vmin+ (Vmax−Vmin)/ [1+ ([I]/IC50)s], and v was determined at 1 mM PEP and 1 mM S3P, with glyphosate concentrations ranging from 0.0001 to 100 mM. (TIF) [file pone.0039579.s002.tif]
